# Alternative Transaxillary Access for Transcatheter Aortic Valve Implantation

**DOI:** 10.3390/jcm14145127

**Published:** 2025-07-18

**Authors:** Konrad Wisniewski, Gerrit Kaleschke, Fernando De-Torres-Alba, Sven Martens, Heinz Deschka

**Affiliations:** 1 Department of Cardiothoracic Surgery, University Hospital Muenster, 48149 Muenster, Germany; sven.martens@ukmuenster.de (S.M.); heinz.deschka@ukmuenster.de (H.D.); 2 Department of Cardiology III-Adult Congenital and Valvular Heart Disease, University Hospital Muenster, 48149 Muenster, Germany; gerrit.kaleschke@ukmuenster.de (G.K.); fernando.detorresalba@ukmuenster.de (F.D.-T.-A.)

**Keywords:** TAVI, transaxillary access, transcatheter aortic valve implantation

## Abstract

**Background/Objectives:** Currently, the transfemoral approach is recognized as the primary method for accessing transcatheter aortic valve implantation (TAVI). However, alternative techniques are needed when the transfemoral access is not suitable. We proposed that a modified transaxillary approach through the distal left axillary artery is both viable and safe for conducting TAVI, potentially offering benefits for patients. **Methods:** From December 2018 to February 2024, a total of 24 patients (7 women, average age 77.9 ± 8 years) received TAVI using transaxillary access via the left axillary artery. The participants suffered from symptomatic severe aortic stenosis and were deemed TAVI candidates with iliofemoral anatomy unsuitable for a transfemoral route. The patient group displayed a high perioperative risk profile, with significant peripheral artery disease or severe obstructive infrarenal aortic conditions. The implantation of the aortic prosthesis was carried out through the left distal axillary artery. A balloon-expandable valve was used in every instance. **Results:** In the examined cohort, the 30-day mortality rate was 4.2%. A new pacemaker was necessary for four patients (16.7%). One case exhibited a new moderate neurological dysfunction. Additionally, one patient required surgical revision of the access point due to ischemia. **Conclusions:** Our findings indicate that transaxillary TAVI via the distal left axillary artery has yielded encouraging outcomes. This approach is practicable and safe, does not prolong the procedure, minimizes surgical trauma, ensures excellent access regardless of chest anatomy, and is sparing for the brachial plexus. As a single-center pilot study, our findings require confirmation in larger, prospective cohorts with extended follow-up to fully validate the safety and long-term efficacy of this technique.

## 1. Introduction

According to the actual ESC/EACTS Guidelines for the management of valvular heart disease, transcatheter aortic valve implantation (TAVI) is the first-line treatment for severe aortic stenosis in patients with higher surgical risk profiles [1]. The transfemoral (TF) route remains the preferred access due to its minimally invasive nature and favorable outcomes. Nevertheless, approximately 10–15% of patients present with severe iliofemoral disease, necessitating alternative access routes, such as the transapical, transaortic, transcarotid, or transaxillary routes [2,3].

Recent comparative studies and meta-analyses have demonstrated that the transaxillary route is non-inferior to transfemoral access with regard to device success and 30-day mortality, with lower morbidity and better recovery profiles compared to other alternative routes such as transapical or transaortic access. It also compares favorably in terms of vascular and neurological complications [2,4,5]. These data reinforce the rationale for prioritizing the axillary approach and its use as the preferred first-choice alternative route when transfemoral access is not feasible.

The transaxillary approach has been established as the first-choice alternative if transfemoral access is not possible. Transaxillary TAVI outcomes are similar to those of transfemoral access regarding success and complication rates. However, the findings on stroke risk show considerable variability [2,3,4].

Conventional transaxillary TAVI is associated with specific anatomical and procedural challenges, including proximity to the brachial plexus, potential difficulties with vascular access due to vessel tortuosity, and suboptimal sheath alignment, which may increase the risk of vascular or neurological complications [3,4,5]. The management of potential complications in conventional transaxillary access can also be challenging. The distal segment of the left axillary artery offers several advantages: it allows a more direct and stable sheath trajectory, is less likely to compromise the brachial plexus, and, when accessed via the medial bicipital groove, provides an optimal entry angle and improved control during device delivery [3,4,6]. The distal left axillary artery was chosen for this modification specifically to mitigate these issues and enhance procedural safety and efficacy.

We present our institutional experience with this modified transaxillary approach via the surgically exposed distal left axillary artery.

## 2. Materials and Methods

### 2.1. Patient Selection

This retrospective analysis included 24 consecutive patients who underwent TAVI via the distal left axillary artery between December 2018 and February 2024. All patients had symptomatic severe aortic stenosis (mean pressure gradient 43.8 ± 10.5 mmHg; effective valve area 0.7 ± 0.2 cm^2^) and were deemed unsuitable for TF access due to peripheral artery disease or infrarenal aortic occlusion.

Preoperative characteristics, periprocedural details, and postoperative outcomes were retrospectively collected from our institutional database. All clinical outcomes were defined according to the updated definitions of the Valve Academic Research Consortium 3 (VARC-3) [7].

Data are presented as numbers and frequencies for categorical variables and as mean ± standard deviation for continuous variables. The statistical analysis was performed using SPSS Statistics v. 28.0 (IBM Corporation, Armonk, NY, USA).

The Ethics Committee of University Hospital Muenster (8 November 2024, diary no. 2024-691-f-S) approved this retrospective study. According to the approval, individual informed consent was not required in this retrospective analysis and was therefore waived.

### 2.2. Preoperative Planning and Operative Technique

**Figure 1:**
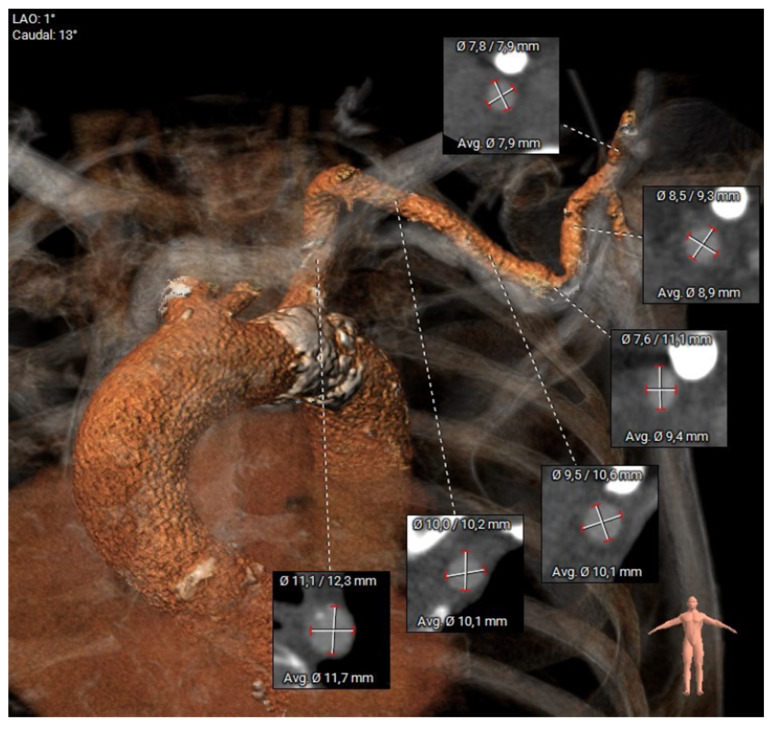
CTA reconstruction of aorta and axillary artery with vessel diameters. Calcification load can be identified.

All patients referred for TAVI underwent standard preprocedural assessment of aortic annulus parameters using MSCT, angiography, and transesophageal echocardiography (TEE). Three-dimensional reconstruction of MSCT images was performed using 3mensio (3mensio Medical Imaging BV, Bilthoven, The Netherlands), allowing for semiautomated assessment of calcification load and supra-aortic vessel diameters (Figure 1).

Transaxillary access for TAVI was considered appropriate if the diameter of the axillary artery in segment 3 (distal) was >6 mm and the potential puncture site was free from calcification. A patient with a patent left internal mammary artery graft after CABG was deemed prohibitive for an ipsilateral transaxillary access.

In the presented patient cohort, only the left axillary artery was used as an access route, although the approach presented can be performed bilaterally.

**Figure 2:**
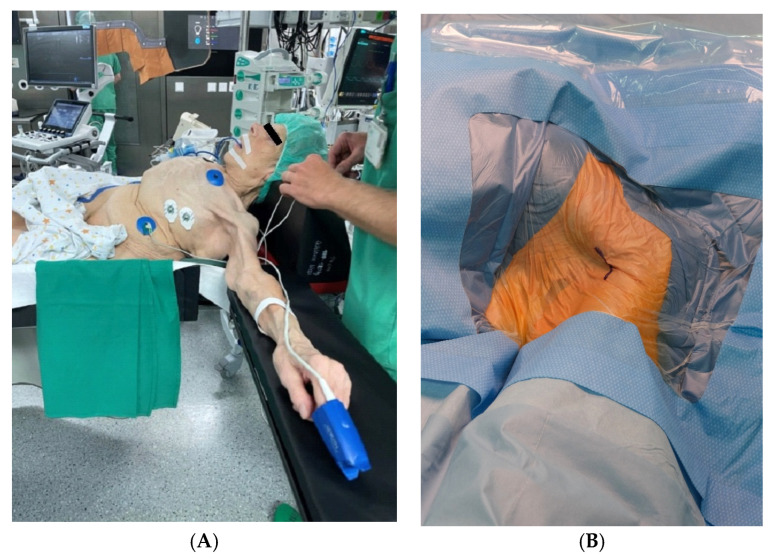
Preoperative photographs (**A**,**B**) of the setting in the hybrid operation theater.

All procedures were performed under general anesthesia in a hybrid operating room (Figure 2) by 3 operators and a scrub nurse. The patients were positioned supine, with the left arm 90 degrees abducted and slightly rotated externally.

To access the distal axillary artery, we performed an approximately 4 cm long skin incision following the lower border of the pectoralis major muscle to the proximal bicipital groove [8].

**Figure 3:**
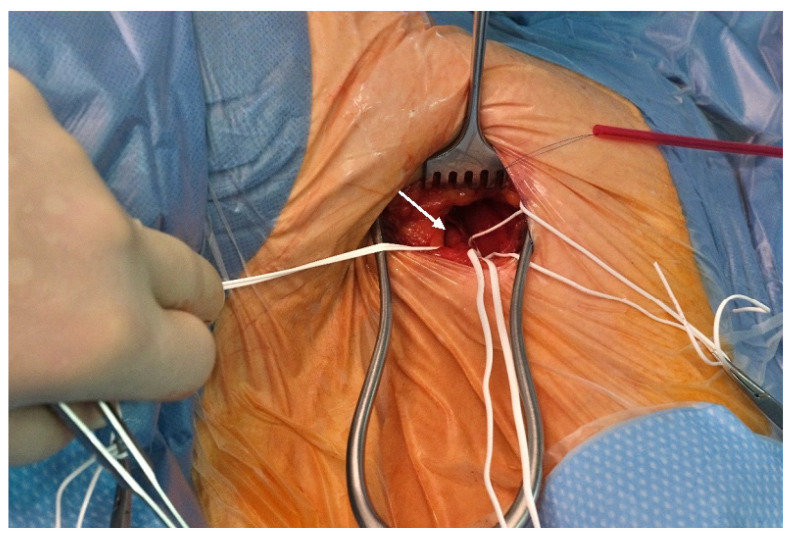
Intraoperative photograph. White arrow points to axillary artery.

After minimal dissection of the subcutaneous tissue (Figure 3), the distal axillary artery was exposed. Special attention was paid to the peripheral branches of the brachial plexus (median, radial, and ulnar nerves), which can be identified easily and surround the vessel laterally, dorsally, and medially.

The axillary artery was secured with a vessel loop, and a purse suture was placed around the puncture site.

After systemic heparinization, the axillary artery was punctured, and a suitable guide wire was introduced using the Seldinger technique.

The further steps corresponded to the standard procedure of TAVI with either balloon-expandable or a self-expanding device.

After removal of the delivery system, the incision of the axillary artery was closed with a purse-string suture after controlling for hemostasis at the puncture site, and a final angiogram was performed to evaluate perfusion in the ipsilateral upper extremity.

Although the described technique is technically feasible for both the left and right axillary arteries, only the left side was utilized in this cohort. This preference was primarily due to the more direct anatomical route to the aortic root from the left, which facilitates optimal sheath alignment and device delivery [3,6]. Additionally, the presence of anatomical variations, such as the course of the subclavian artery and arch anatomy, influenced decision-making. Operator familiarity and workflow standardization also contributed.

It is strongly advised to avoid implanting self-expandable prostheses from the right side. The angle at which the system enters the aortic root is typically very steep, and it approaches from the inner curvature. This starting point for implantation is unfavorable, making the perfect positioning of the transcatheter valves challenging. Self-expanding transcatheter heart valves such as the SAPIEN 3 valve prove beneficial in this scenario, as their balloon expansion aids in achieving better axial alignment [6,9].

Right transaxillary access also encounters specific challenges because of the tortuous and angled path between the right axillary artery and the aortic root, which includes the double curves of the innominate artery and the ascending aorta [9].

While bilateral approaches are reported, most published series favor the left side, and available data do not indicate significant differences in safety or procedural outcomes between left and right axillary access, provided that careful preoperative imaging and planning are performed [5,6,10]. Further comparative research on laterality may clarify any subtle differences.

## 3. Results

### 3.1. Baseline Characteristics

**Figure 4:**
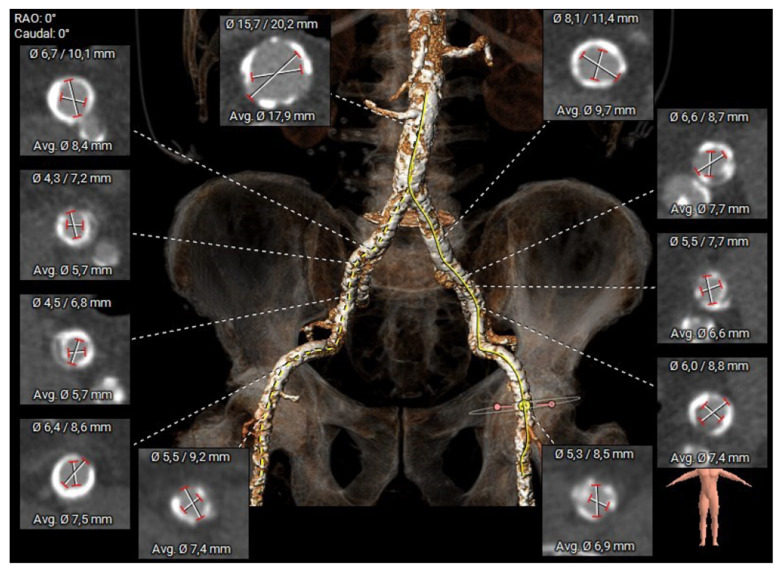
CTA reconstruction of femoral arteries. Calcification load is prohibitive for transfemoral TAVI.

The baseline characteristics of the study cohort are presented in Table 1. The cohort included 17 men and 7 women, with a mean age of 77.9 ± 8 years. The mean EuroScore II was 7.4 ± 4.7%, indicating an intermediate to high surgical risk. The patient cohort showed severe peripheral artery disease (n = 16, 66.7% of cases) or severe occluding infrarenal aortic disease in the remaining cases (n = 8) (Figure 4). Severe renal impairment was present in 12.5%, and diabetes in 29.2%. The mean left ventricular ejection fraction was 52.3 ± 11.5%, and the mean aortic valve area was 0.7 ± 0.2 cm^2^. A history of stroke or TIA was presented in 20.8% of the patients.

This cohort reflects the typical alternative-access TAVI population: elderly, high-risk, and extensive comorbidities precluding transfemoral access, consistent with large registries and recent reviews.

### 3.2. Periprocedural Outcomes

The periprocedural data are displayed in Table 2. In all cases, a balloon-expandable valve with a diameter of 25.6 ± 2.4 mm was implanted (Sapien 3). The mean procedure time was 102.6 ± 46 min, and the average fluoroscopy time was 20.8 ± 14.7 min. The average contrast volume was 110.1 ± 42.0 mL. Pre-dilatation was performed in 54.2% and post-dilatation in 20.8%. No conversion to another access route was required.

Table 3 presents clinical data of our patient collective. In-hospital mortality (30 days) was 4.15%, caused by one lethal complication due to aspiration, consecutive hypoxia, and pulseless electrical activity on postoperative day 1. Permanent pacemaker implantation was required in four patients (16.7%). One patient experienced moderate stroke-related disability (mRS). One patient (4.2%) experienced perioperative myocardial infarction with stenting. Major vascular access complications occurred in one case (4.2%), requiring surgical revision due to ischemia. Three patients (12.5%) required transfusions (major bleeding, Type 3) due to gastrointestinal causes. No access-related bleeding has occurred. Technical success (at exit from the procedure room) was 100%. Device success was observed in 91.7% (22/24 patients; one mortal event and one vascular complication) of all cases (VARC-3 Criteria) [7]. No moderate or severe prosthetic aortic valve regurgitation was observed at discharge, and mean aortic gradients decreased from 43.8 ± 10.5 mmHg to 13.0 ± 3.1 mmHg. The mean length of hospital stay was 7.3 ± 5.4 days.

## 4. Discussion

### 4.1. Interpretation of the Results

Transaxillary access is a feasible and safe first-choice alternative for TAVI in patients with challenging anatomy for femoral access. Our findings support the increasing evidence that transaxillary access, especially through the distal left axillary artery, yields outcomes comparable to those of large registry data [4,5].

Even with a relatively small group of 24 participants, we achieved a high rate of procedural success and a low rate of serious complications, aligning with larger registry studies [4,8,9]. Our results are in line with other studies focusing on transaxillary procedures, where device success usually ranges from 92% to 97%, and conversion rates remain low. Multiple reviews and detailed analyses support that transaxillary access is just as effective as transfemoral access when it comes to device success and 30-day mortality, with comparable risks of major vascular and bleeding issues [7,10].

Our reported mortality rate is comparable to published data for transaxillary TAVI in high-risk patients, where 30-day mortality ranges from 4% to 6% [4,10]. Complication rates align with other balloon-expandable valve series using alternative access, reflecting both patient risk profile and device type [10,11]. Stroke or neurological dysfunction is similar to some reports but remains within the range of single-center experiences. Notably, there were no disabling strokes [8,9]. Major vascular complications (4.2%) and bleeding (12.5%) fall within the reported ranges for transaxillary access (vascular: 2–6%, bleeding: 10–15%) [4,12]. The excellent hemodynamic results (no moderate/severe regurgitation, low gradients at discharge) demonstrate the procedural efficacy of the axillary approach.

As observed in our series, even though a surgical cut-down was performed, the intervention time and fluoroscopy duration were not significantly prolonged compared to TF-TAVI benchmarks. The literature also highlights a significant learning curve, with further reductions in complications as operator experience grows [11,13].

Furthermore, device success in our study (91.7%) compares to the outcomes reported in the TVT Registry, where TAx access showed a success rate of 97.3% [10]. This reinforces the technical reliability of this access route when proper preoperative imaging and surgical planning are in place. Technical success (at exit from the procedure room) was 100%.

A notable finding in our cohort was the relatively high rate of new permanent pacemaker implantation (16.7%). This aligns with previous reports suggesting higher conduction disturbance risk when balloon-expandable valves are used in alternative accesses [11,13]. Nevertheless, the described access is also suitable for self-expanding devices.

The exclusive use of balloon-expandable valves may partially explain the relatively high rate of new permanent pacemaker implantation in our cohort. It is notable and likely multifactorial. In addition to the recognized association with balloon-expandable valves, several well-established risk factors should be considered.

Specifically, individual implantation depth is a significant procedural predictor of post-TAVI conduction disturbances and permanent pacemaker requirement. This relationship has been consistently demonstrated across different valve types, including balloon-expandable devices. In our study, systematic measurement of implantation depth was not performed. However, it is plausible that suboptimal depth contributed in some cases [14].

Baseline conduction disturbances, such as pre-existing right bundle branch block or first-degree AV block, on the baseline ECG are also strong predictors of post-interventional pacemaker implantation. In our study, although systematic data on baseline conduction status and implantation depth were not collected for all cases, several patients had pre-existing right bundle branch block or borderline conduction intervals on preoperative ECGs [14,15].

Also, different anatomical features such as increased septal wall thickness, heavy calcification of the left ventricular outflow tract, short membranous septum, and lower left coronary cusp height could increase the risk of conduction system injury during TAVI [14,16].

Our retrospective and relatively small cohort did not allow for detailed systematic analysis of all these parameters, which we acknowledge as a limitation. Future studies should include prospective evaluation of implantation depth, baseline conduction status, and anatomical risk factors to clarify predictors of pacemaker requirement after transaxillary TAVI.

Neurological events, particularly stroke and transient ischemic attacks, remain a concern in TAx-TAVI. In our cohort, only one case (4.15%) of moderate neurological deficit was observed, which is below the stroke incidence reported in some larger studies (up to 6.3%) [10]. Using the left axillary artery, located further from the aortic arch, may reduce the risk of cerebral embolization compared to subclavian access [6,12].

Notably, the need for surgical revision at the access site was minimal without access-related major bleeding events, suggesting that with careful selection and vascular preparation, complications such as bleeding and ischemia can be effectively prevented [5,11,12].

### 4.2. Benefits of the Surgical Approach

The axillary approach is favored as the first-choice alternative route for TAVI when transfemoral access is not feasible, due to its peripheral accessibility, favorable sheath trajectory, and reduced invasiveness compared to thoracic access routes [2,4,5].

Transaxillary access provides several advantages over other alternative routes, such as transapical or transaortic approaches, which have been associated with higher morbidity, mortality, and prolonged recovery [2,6]. In contrast, the transaxillary route is less invasive, does not require thoracotomy, and allows a percutaneous or minimal surgical approach [3,12].

Although the transcarotid approach has gained popularity in recent years, it may carry a higher risk of neurological complications in certain populations, and operator experience with axillary access is often more widespread [4,5,6]. Comparative studies consistently demonstrate that axillary access yields equivalent or superior outcomes with respect to procedural success, safety, and recovery time, thereby supporting its role as the preferred alternative route.

We believe this modified transaxillary approach has advantages over the classic subclavian approach [4]. The axillary artery is a particularly attractive option due to its peripheral location, relatively straight course, and the ability to access it without entering the thoracic cavity.

Notably, the main procedural advantage of this approach consists of an easy and relatively atraumatic surgical access, especially in obese patients or patients with an unfavorable shape of the chest.

The optimal entry angle and the longer intravascular course of the sheath, regardless of chest anatomy, lead to better guidance and greater stability of the delivery system, which facilitates device deployment and simple management of any potential complications.

From a practical standpoint, the distal part of the left axillary artery provides stable access and excellent exposure without compromising the brachial plexus. Our surgical approach minimized nerve injury risk, a complication previously reported in up to 10% of TAx-TAVI patients [5].

The axillary approach is favored among alternative routes due to peripheral accessibility and the avoidance of thoracotomy. It leads to quicker recovery and shorter hospital stays compared to transapical/direct aortic approaches [2,3].

Our approach is far less complex than direct percutaneous access [9,13]. The axillary vessel is directly accessible, making complication management much easier. The escalation to a complex procedure involving preparation for stenting at the implantation site and the use of expensive materials, as is the case with percutaneous access, is not necessary. If necessary, vessel repair can be performed at any time without escalation. The procedure time is comparable, but contrast agent usage is lower in our case

Considering possible points for improvement, a transition from general anesthesia to regional anesthesia is feasible and would provide additional benefits.

The main limitation of this approach is the requirement for a minimum vessel diameter of 6 mm, which may exclude some patients from eligibility (Figure 4).

It should be emphasized that this investigation represents a pilot study with a limited number of patients. While our outcomes are promising and align with existing registry data, further multicenter and prospective studies with larger patient populations and longer follow-up are essential to confirm these results and determine the durability and reproducibility of the modified transaxillary approach.

## 5. Conclusions

Our experience shows that transaxillary TAVI via the distal left axillary artery has shown promising results for high-risk patients with contraindications to transfemoral access. This study supports the feasibility and safety of transaxillary TAVI. Key procedural advantages include easy access, no risk to the brachial plexus, optimal sheath entry angle, better and more stable guidance of the delivery system, and straightforward complication management. This access does not prolong the intervention time, minimizes surgical trauma, provides excellent exposure independent of the chest anatomy without intrathoracic dissection, and is sparing for the brachial plexus. The main procedural limitation is the need for a vessel diameter greater than 6 mm to ensure safety and technical feasibility. The clinical outcomes, including device success, mortality, and complication rates, closely match contemporary series and recent meta-analyses, supporting the axillary route as a preferred alternative access for TAVI. The presented technique is a promising alternative for patients with challenging peripheral vascular anatomy and should be considered in centers experienced in vascular surgery. Neurological and vascular complications were minimal and in line with larger registry reports. Additionally, regional anesthesia is an option in this situation and offers several benefits.

Given the small sample size, our study should be regarded as a pilot evaluation, and future larger-scale, prospective investigations are warranted to confirm these initial results and assess long-term clinical outcomes.

### 5.1. Limitations

While our results are promising, this study is limited by its retrospective, single-center design and relatively small sample size. A control group using standard TF access was not available, limiting direct comparison. Nonetheless, the consistency of our outcomes with larger registries supports the external validity of our observations [4,10].

### 5.2. Future Directions

Future multicenter studies comparing percutaneous versus surgical TAx approaches and balloon-expandable versus self-expanding valves are warranted. We will attempt to apply regional anesthesia as a possible option, which we think would provide additional benefits.

Our findings were presented as a short communication titled “The Alternative Transaxillary Access for Transcatheter Aortic Valve Implantation” at the 54th Annual Congress of the German Society for Thoracic and Cardiovascular Surgery (DGTHG), held in Hamburg from 15 to 17 February 2025 [17].

## Figures and Tables

**Table 1:** Baseline characteristics of the study cohort.

Baseline Characteristics	Overall N = 24 (%, Mean ± SD)
Age (years)	77.9 ± 8
Sex (female)	7 (29.2%)
Weight (kg)	74.7 ± 12.8
Height (cm)	171.0 ± 12.2
BMI	25.4 ± 3.5
EuroScore II (%)	7.4 ± 4.7
Myocardial infarction	3 (12.5%)
Prior PCI	4 (16.7%)
Prior pacemaker	2 (8.3%)
Prior stroke/TIA	5 (20.8%)
History of smoking	9 (37.5%)
Diabetes mellitus on insulin	7 (29.2%)
Chronic pulmonary disease	7 (29.2%)
Arterial hypertension	18 (75%)
Peripheral vascular disease	16 (66.7%)
Severe renal impairment (GFR < 30 mL/min/1.73 m^2^)	3 (12.5%)
Creatinine (mg/dL)	1.2 ± 0.8
Malignant disease	4 (16.7%)
Atrial fibrillation	8 (33.3%)
**Echocardiography**	
Left-ventricular ejection fraction (%)	52.3 ± 11.5
Aortic valve area (cm^2^)	0.7 ± 0.2
Mean aortic valve gradient (mmHg)	43.8 ± 10.5
Peak aortic valve gradient (mmHg)	73.3 ± 15.5

BMI—body mass index; PCI—percutaneous coronary intervention; TIA—transient ischemic attack; GFR—glomerular filtration rate.

**Table 2:** Periprocedural details.

Periprocedural Details	Overall N = 24 (%, Mean ± SD)
Procedure time (min)	102.6 ± 46.1
Fluoroscopy time (min)	20.8 ± 14.7
Contrast medium (mL)	110.1 ± 42.0
Pre-dilatation	13 (54.2%)
Post dilatation	5 (20.8%)
Sapien3	24 (100%)
Valve diameter (mm/mean)	25.6 ± 2.4
**Valve size (mm)**	
23	9
26	9
29	6

**Table 3:** Clinical outcomes of patients undergoing transaxillary transcatheter aortic valve implantation.

Clinical Outcomes	Overall N = 24 (%, Mean ± SD)
In-hospital-death	1 (4.15%)
Device Intervention success	22 (91.7%)
Conversion to surgery	0 (0%)
Myocardial infarction	1 (4.15%)
Stroke (moderate disability/Rankin)	1 (4.15%)
New permanent pacemaker	4 (16.7%)
Major bleeding (Type 3, gastrointestinal)	3 (12.5%)
Major vascular complications	1 (4.15%)
Acute kidney injury (>AKI Stage 2)	2 (8.3%)
Dialysis	1 (4.15%)
Mean aortic valve gradient (mmHg) (at discharge)	13.0 ± 3.1
Peak aortic valve gradient (mmHg) (at discharge)	22.9 ± 3.4
Length of hospital stay (days)	7.3 ± 5.4

## Data Availability

The original contributions presented in this study are included in the article. Further inquiries can be directed to the corresponding author.

## Abbreviations

AKI	Acute Kidney Injury
BMI	Body Mass Index
CT	Computed Tomography
EUROSCORE II	European System for Cardiac Operative Risk Evaluation II
GFR	Glomerular Filtration Rate
mRS	Modified Rankin Scale
MSCT	Multislice Computed Tomography
PCI	Percutaneous Coronary Intervention
TAVI	Transcatheter Aortic Valve Implantation
TAx	Transaxillary
TIA	Transient Ischemic Attack
TF	Transfemoral
VARC	Valve Academic Research Consortium

## References

[1] Vahanian A., Beyersdorf F., Praz F., Milojevic M., Baldus S., Bauersachs J., Capodanno D., Conradi L., De Bonis M., De Paulis R. (2022). 2021 ESC/EACTS Guidelines for the management of valvular heart disease: Developed by the Task Force for the management of valvular heart disease of the European Society of Cardiology (ESC) and the European Association for Cardio-Thoracic Surgery (EACTS). Rev. Esp. Cardiol. Engl. Ed..

[2] Abdelnour M.W., Patel V., Patel P.M., Kasel A.M., Frangieh A.H. (2024). Alternative access in transcatheter aortic valve replacement—An updated focused review. Front. Cardiovasc. Med..

[3] Harloff M.T., Percy E.D., Hirji S.A., Yazdchi F., Shim H., Chowdhury M., Malarczyk A.A., Sobieszczyk P.S., Sabe A.A., Shah P.B. (2020). A step-by-step guide to trans-axillary transcatheter aortic valve replacement. Ann. Cardiothorac. Surg..

[4] Herremans A., Stevesyns D.T., El Jattari H., Rosseel M., Rosseel L. (2023). The Place of Transaxillary Access in Transcatheter Aortic Valve Implantation (TAVI) Compared to Alternative Routes—A Systematic Review Article. Rev. Cardiovasc. Med..

[5] Van Wely M., Van Nieuwkerk A.C., Rooijakkers M., Van Der Wulp K., Gehlmann H., Verkroost M., Van Garsse L., Geuzebroek G., Baz J.A., Tchétché D. (2024). Transaxillary versus transfemoral access as default access in TAVI: A propensity matched analysis. Int. J. Cardiol..

[6] Sündermann S.H., Dreger H., Hinkov H., Kempfert J. (2023). The 10 Commandments for Transaxillary TAVI. Innov. Technol. Tech. Cardiothorac. Vasc. Surg..

[7] Généreux P., Piazza N., Alu M.C., Nazif T., Hahn R.T., Pibarot P., Bax J.J., Leipsic J.A., Blanke P., Blackstone E.H. (2021). Valve Academic Research Consortium 3: Updated Endpoint Definitions for Aortic Valve Clinical Research. J. Am. Coll. Cardiol..

[8] Ascher E., Haimovici H. (2012). Haimovici’s Vascular Surgery.

[9] McGrath D., Lee H., Sun C., Kawabori M., Zhan Y. (2024). Right transaxillary transcatheter aortic valve replacement is comparable to left despite challenges. Gen. Thorac. Cardiovasc. Surg..

[10] Dahle T.G., Kaneko T., McCabe J.M. (2019). Outcomes Following Subclavian and Axillary Artery Access for Transcatheter Aortic Valve Replacement. JACC Cardiovasc. Interv..

[11] Schäfer U., Deuschl F., Schofer N., Frerker C., Schmidt T., Kuck K.H., Kreidel F., Schirmer J., Mizote I., Reichenspurner H. (2017). Safety and efficacy of the percutaneous transaxillary access for transcatheter aortic valve implantation using various transcatheter heart valves in 100 consecutive patients. Int. J. Cardiol..

[12] Sugiura A., Sudo M., Al-Kassou B., Shamekhi J., Silaschi M., Wilde N., Sedaghat A., Becher U.M., Weber M., Sinning J.-M. (2022). Percutaneous trans-axilla transcatheter aortic valve replacement. Heart Vessels.

[13] Muscoli S., Cammalleri V., Bonanni M., Prandi F.R., Sanseviero A., Massaro G., Di Luozzo M., Chiocchi M., Ascoli Marchetti A., Ippoliti A. (2022). The Transaxillary Route as a Second Access Option in TAVI Procedures: Experience of a Single Centre. Int. J. Environ. Res. Public Health.

[14] Mauri V., Reimann A., Stern D., Scherner M., Kuhn E., Rudolph V., Rosenkranz S., Eghbalzadeh K., Friedrichs K., Wahlers T. (2016). Predictors of Permanent Pacemaker Implantation After Transcatheter Aortic Valve Replacement With the SAPIEN 3. JACC Cardiovasc. Interv..

[15] Bruno F., D’Ascenzo F., Vaira M.P., Elia E., Omedè P., Kodali S., Barbanti M., Rodès-Cabau J., Husser O., Sossalla S. (2021). Predictors of pacemaker implantation after transcatheter aortic valve implantation according to kind of prosthesis and risk profile: A systematic review and contemporary meta-analysis. Eur. Heart J.-Qual. Care Clin. Outcomes.

[16] Hokken T.W., Muhemin M., Okuno T., Veulemans V., Lopes B.B., Beneduce A., Vittorio R., Ooms J.F., Adrichem R., Neleman T. (2022). Impact of membranous septum length on pacemaker need with different transcatheter aortic valve replacement systems: The INTERSECT registry. J. Cardiovasc. Comput. Tomogr..

[17] Wisniewski K., Kaleschke G., De Torres-Alba F., Martens S., Deschka H. (2025). The Alternative Transaxillary Access for Transcatheter Aortic Valve Implantation. Proceedings of the 54th annual congress of the German Society for Thoracic and Cardiovascular Surgery (DGTHG).

